# Improving Our Risk Communication: Standardized Risk Levels for Brief Assessment of Recidivism Risk-2002R

**DOI:** 10.1177/10790632211047185

**Published:** 2021-10-20

**Authors:** Julie Blais, Kelly M. Babchishin, R. Karl Hanson

**Affiliations:** 1 Department of Psychology and Neuroscience, 3688 Dalhousie University, Halifax, NS, Canada; 2 Department of Psychology, 6339 Carleton University, Ottawa, ON, Canada; 3 Mental Health Research Institute, Royal Ottawa Mental Health Centre, University of Ottawa, Ottawa, ON, Canada

**Keywords:** risk communication, BARR-2002R, risk, recidivism, offending

## Abstract

A Five-Level Risk and Needs system has been proposed as a common language for standardizing the meaning of risk levels across risk/need tools used in corrections. Study 1 examined whether the Five-Levels could be applied to BARR-2002R (*N* = 2,390), an actuarial tool for general recidivism. Study 2 examined the construct validity of BARR-2002R risk levels in two samples of individuals with a history of sexual offending (*N* = 1,081). Study 1 found reasonable correspondence between BARR-2002R scores and four of the five standardized risk levels (no Level V). Study 2 found that the profiles of individuals in Levels II, III, and IV were mostly consistent with expectations; however, individuals in the lowest risk level (Level I) had more criminogenic needs than expected based on the original descriptions of the Five-Levels. The Five-Level system was mostly successful when applied to BARR-2002R. Revisions to this system, or the inclusion of putatively dynamic risk factors and protective factors, may be required to improve alignment with the information provided by certain risk tools.

## Introduction

Risk assessment is a ubiquitous practice with a long history in both the criminal justice and forensic mental health systems (Litwack et al., 2006; Monahan et al., 2001). It allows for evidence-based responses, informing decisions such as classifications within institutions (Hilton et al., 2016), whether someone should be incarcerated indefinitely (Blais & Forth, 2014; Jackson & Hess, 2007), and, if released into the community, how they should be supervised and managed (Babchishin & Hanson, 2020; Douglas et al., 2013). There have been advancements in risk assessment, such as moving from unstructured clinical judgment to utilizing structured and empirically supported risk instruments (Grove et al., 2000; Hanson & Morton-Bourgon, 2009); however, similar improvements are not as evident in communicating risk to decision makers. This lack of progress is especially concerning considering that risk information provided by evaluators is heavily weighted by decision makers and predictive of ultimate outcomes (Blais, 2015; Hilton et al., 2016). The most valid and reliable assessment of risk becomes meaningless if decision makers misinterpret the results (Heilbrun et al., 1999). A better standard of risk communication is required.

### Risk Communication

Although existing guidelines provide some direction for forensic evaluators (American Psychological Association, 2013), there are no specific rules governing how risk assessment should be implemented and no formal body overseeing its proper administration (Heilbrun & Brooks, 2010). It is, therefore, left to the evaluator to determine best practices in making important decisions, such as which tool or tools to utilize, what information to include, and how best to integrate and communicate this information into a practical and cohesive report. Every decision or judgment call increases the chances that bias and inconsistencies will be introduced into the assessment (Zapf & Dror, 2017). Risk communication literature has tended to focus on identifying limitations and not necessarily on innovating potential solutions. Such limitations include an overwhelming preference for categorical terminology (e.g., low, moderate, and high) among practitioners and decision makers (Blais & Forth, 2014; Evans & Salekin, 2016) despite little agreement on the meaning and boundaries of each risk category (Hilton et al., 2008; Scurich, 2018). By using similar words, risk communication may appear to be standardized; however, the categories themselves are not actually standardized because they do not have the same meaning across evaluations. The lack of consensus results in drastically different perceptions of risk for individual cases (Batastini et al., 2019; Krauss et al., 2018).

There are also dozens of risk assessment scales, each with their own strengths and limitations, available for assessing the likelihood of diverse reoffending outcomes (Singh et al., 2014). Even among scales designed to predict the same outcome, there are fundamental differences in the factors that they comprise, the risk estimates associated with each score, and the language used to communicate risk (Barbaree et al., 2006; Jung et al., 2013). The existence of multiple risk tools raises questions about the consistency of the information provided by each tool. Barbaree et al. (2006) examined the extent of incongruence among five commonly used actuarial measures for predicting sexual recidivism (Rapid Risk Assessment for Sexual Offense Recidivism [RRASOR; Hanson, 1997], Static-99 [Hanson & Thornton, 1999, 2000], Violence Risk Appraisal Guide [VRAG; Quinsey et al., 1998], Sex Offender Risk Appraisal Guide [SORAG; Quinsey et al., 1998], and Minnesota Sex Offender Screening Tool-Revised [MnSOST-R; Epperson et al., 1998]) for 468 individuals with a history of sexual offending being assessed for treatment at the Warkworth Sexual Behaviour Clinic. Using percentiles to rank the risk of individuals, results indicated large variability with fewer than 5.0% of individuals consistently rated as either high risk or low risk across all five scales. Jung et al. (2013) reported similar results among several of the same actuarial tools (Static-99R [Hanson & Thornton, 2000; Helmus et al., 2012; *n* = 361], Static-2002R [Hanson & Thornton, 2003; Helmus et al., 2012; *n* = 345], and SORAG [*n* = 82]) and the Sexual Violence Risk-20 (SVR-20; Boer et al., 1997; *n* = 74) for a sample of individuals from a forensic psychiatric facility. Overall percentage agreement in assigning individuals to risk categories ranged from 23.2% (between Static-99R and SVR-20) to 71.4% (between SORAG and SVR-20; Median agreement = 46.7%).

Babchishin et al. (2012) combined information from several samples (*k* = 20, *N* = 7,491) to examine different methods of combining risk scale scores from Static-99R, Static-2002R, and RRASOR (lowest, highest, and average). In order to compare different rules for combining risk scales, the scales were standardized to a common metric (i.e., hazard ratio). Averaging the hazard ratios produced better predictive accuracy (AUC = .69) than choosing the highest score (AUC = .68), and produced better calibration, defined as the match between expected recidivism rates from the tool’s norms and observed recidivism rates, than either choosing the lowest or highest risk score (also replicated by Lehmann et al., 2013). In short, combining risk tools using scale-specific categorizations is not optimal. Instead, standardizing the meaning of risk category labels across risk tools could facilitate consistency across risk tools and thereby allow individuals with similar risk to receive similar correctional responses irrespective of the risk tools being used.

### Developing a Common Language

Considering current risk communication limitations and key advancements in the science of risk assessment, the practices of risk communication could benefit from specific guidelines. Risk communication could be advanced by (a) providing non-arbitrary anchors to commonly used risk labels, (b) increasing consistency in how information from several scales is combined and communicated, and (c) emphasizing both the evaluation of recidivism risk as well as the treatment and management needs of the individual. The Justice Centre’s Five-Level Risk and Needs System (Hanson et al., 2017b) represents a comprehensive attempt at advancing risk communication in corrections and forensic mental health for general offending. Based on research and discussions with clinicians, researchers, correctional practitioners, and decision makers, this system aims to increase the consistency by which risk assessment information is communicated and provide guidance on treatment targets and the appropriate level of programming and supervision, regardless of the risk tool used or jurisdiction or setting. Importantly, the authors of the Five-Levels asserted that these levels can be applied to total scores of existing risk assessment scales. The current study tested the feasibility of this assumption.

The Five-Levels were rooted within the well-established Risk, Need, and Responsivity (RNR) principles of correctional rehabilitation (Andrews & Bonta, 2010) and were intended to provide the necessary information for meeting current best practices guidelines for assessments of general (or any) recidivism. A summary table of the Five-Levels is presented in Appendix 1. Each level is associated with a set of features relevant to the intervention and risk management of individuals at risk for crime, including statistical information in the form of the probability of general recidivism (conviction) within 2 years. For example, Level I encompasses individuals with a less than 5.0% risk of any reoffending after 2 years, while Level V encompasses individuals with the highest risk defined as an 85.0% risk or higher. The levels also provide information on the expected number and type of criminogenic needs (i.e., factors empirically related to recidivism). Inter-individual variation on several different domains, such as psychological factors specific to the individual, interpersonal factors describing prosocial and antisocial relationships, and lifestyle factors that could act as barriers to reintegration, is expected based on assigned risk levels. Finally, the levels also provide a recommended correctional response including placement in secure settings (or not), treatment dosage and prognosis, and level of supervision required to effectively manage risk. For example, individuals placed within Level III would be expected to have multiple criminogenic needs, require considerable monitoring in order to ensure supervision compliance, and would benefit from significant correctional treatment defined as a dosage of 100–200 h. This individual’s risk, however, would also be expected to be meaningfully reduced following treatment with their overall risk level being lowered to Level II (Hanson et al., 2017b). Note that these level descriptions are for general (any) recidivism; separate levels have been developed and validated for assessing the risk of sexual recidivism (e.g., Hanson et al., 2017a).

In terms of its implementation, the authors of the Five-Levels have proposed heuristics for estimating risk-level membership based on empirically derived actuarial risk tools (i.e., risk tools for which a range of scores are associated with recidivism rate estimates; Hanson et al., 2017a, 2017b). There are specific recommendations on the amount and quality of data required for deriving the Five-Levels for risk assessment scale (see Hanson et al. (2017a) for a full example of applying the risk levels). First, individual scores for a large sample representative of the desired population are needed containing 500–1,000 individuals, 100 of which are recidivists. The data must also contain a follow-up period of at least 2-years (a longer follow-up of at least 5 years is recommended when estimating sexual recidivism risk; Hanson et al., 2017a). From these data, 2-year expected general recidivism rates, percentile ranks, and odds ratios are calculated in order to define the boundaries of the Five-Levels. Note that different datasets can be used to estimate different parameters. For example, a large, routine sample could be used to estimate the percentile ranks, and different samples could be used to estimate recidivism base rates and the change in relative risk based on risk scores (odds or hazard ratios).

The Five-Level system was designed for describing the risk and needs of individuals at risk for general recidivism and has been applied to instruments designed for that purpose (e.g., LSI-R; Andrews & Bonta, 2001; see Kroner et al., 2020). For example, Kroner et al. (2020) applied the Five-levels to the LSI-R in a large sample of 24,936 individuals on community release. Given that this was a lower risk sample, Level V (with expected general recidivism rates over 85%) was not populated; instead, Level IV was divided into Level IVa and Level IVb (consistent with Hanson et al., 2017a). When compared to a higher risk sample (*N* = 36,303), more individuals in the lower risk community sample were categorized into the lower risk levels of the Five-Levels. The recidivism estimates of the Five-Levels were also closer to the observed base rates of recidivism than the original LSI-R estimates.

The Five-Levels have also been applied to risk tools designed to assess the risk of sexual recidivism, such as Static-99R (see Hanson et al., 2017a), Static-2002R (see Hanson et al., 2017a), STABLE-2007 (Hanson et al., 2007; see Brankley et al., 2017), and the Violence Risk Scale-Sexual Offence version (Wong et al., 2003-2017; see Olver et al., 2018, 2020). In the first application examining sexual recidivism, Hanson et al. (2017a) developed the Five-Levels for both Static-99R and Static-2002R. Given that the expected base rate for Level V (85%) was not observed across the samples, Level IV was divided into Level IVa (Above Average Risk) and Level IVb (Well Above Average Risk), representing individuals at the higher end of the risk distribution for both Static-99R and Static-2002R. The newly created Five-Levels increased the concordance rate for both scales when compared to the original risk categories. In other words, a person was more likely to be classified in the same risk category for both Static-99R and Static-2002R using the Five-Levels compared to the original risk levels.

Although research continues on how best to align the output of existing risk tools with the Five-Levels, the existing studies generally support the feasibility of this approach. Specifically, risk levels based on the standardized Five-Levels are more likely to support the same inferences across measures than whatever inferences would have been implied by the original risk levels of the different measures (e.g., higher concordance between Static-99R and Static-2002R categories using the Five-Levels will result in more consistent decisions; Hanson et al., 2017a). Assessing concordance, however, is only one aspect of validating this nosology. Each level describes the expected risk and treatment profile of an individual placed in that category (see Appendix). It is therefore necessary to validate the accuracy of these profiles; if the individuals within each level match the specific profile of that level, these findings could be used to support the construct validity of the Five-Levels.

### The Current Study

Continuing this program of research, we explored the utility of the Five-Level system for describing the risk for general recidivism based on the Brief Assessment of Recidivism Risk-2002R (BARR-2002; Babchishin et al., 2016), a risk tool derived from Static-2002R items. Static-2002R was originally designed to assess the risk of sexual recidivism and violent recidivism (Hanson & Thornton, 2003) for individuals with a history of sexual offending. Subsequent research, however, indicated that violent and general recidivism were more strongly associated with a subset of Static-2002R items than the total score (Babchishin et al., 2016). Assessing the risk of general recidivism is important for individuals with a history of sexual crime because they are more likely to reoffend with a nonsexual crime than a sexual crime (Hanson & Bussière, 1998; Prentky et al., 1997). Consequently, it should be possible to assign individuals with a history of sexual offending to risk levels that have the same meaning as the original Five-Level system. This assumption, however, requires verification. Specifically, we examined the feasibility of assigning individuals with sexually motivated offenses to the Five-Levels for risk of general recidivism (Study 1) and the extent to which risk level membership had the intended meanings (i.e., construct validity; Study 2).

We chose not to examine a separate violent recidivism outcome given that there exists uncertainty about how best to align the Five-Levels for violent recidivism (e.g., Davies et al., 2020). For example, outstanding issues include identifying the appropriate population, defining what is meant by violence, and determining the required length of follow-up. Furthermore, predicting general recidivism among men with a history of sexual offending is an interesting test of the Five-Level system. Even though there are some unique sex crime specific risk factors (emotional congruence with children and deviant sexual interests), the model proposes that the process of general recidivism is common across diverse populations. Consequently, we expected that the Five-Level system would reasonably describe this group, even if general recidivism was not the primary presenting problem. Although several numerical indicators are used to group scale scores into the Five-Levels, each level has meaning beyond the numerical indicators. For example, someone belonging to Level I (Below Average Risk), despite being assigned based on a numerical probability of recidivism, would be expected to have few risk factors, good treatment prognosis, and little problems with supervision. Therefore, evidence of construct validity for the levels would be achieved by demonstrating that the presence of risk and treatment relevant variables for each level were actually consistent with descriptions provided by the original Five-Level system (see Appendix for overview). In this way, we are providing a test of the congruence between the Five-Level system and the Risk and Need principles of the RNR model. As risk levels increase, so should the number and severity of criminogenic needs; this would indicate that higher risk individuals, classified by the Five-Level system, would require more rehabilitation efforts than individuals classified in the lower levels.

## Study 1

In Study 1, we examined the ability of BARR-2002R scores to identify groups of individuals who resembled the risk levels posited by the Justice Center’s Five-Level Risk and Needs System (Hanson et al., 2017b).

### Method

#### Samples

We used four samples from BARR-2002R development datasets of routine, unselected samples of individuals with a history of sexual offending that had BARR-2002R scores (*N* = 2,390): individuals in federal Canadian corrections from British Columbia (Boer, 2003), individuals in federal Canadian corrections from Quebec (Bigras, 2007), Dynamic Supervision Project (Canada; Hanson et al., 2007, 2015), and German individuals reported to police and ultimately convicted of sexual offenses (Lehmann et al., 2013). Table 1 provides a summary of the samples.

**Table 1.** Description of Samples Included in Study 1 and Study 2.

Sample	Description	*N*	Country	BARR-2002R	Age at Release
				*M* (*SD*)	*M* (*SD*)
Study 1					
Boer (2003)	Archival data from the Offender Management System (OMS) maintained by Correctional Service Canada (CSC) were used to identify all male individuals serving a federal sentence for a sexual offense in British Columbia whose Warrant Expiry Date (WED; the end of their sentence) was between January 1990 and May 1994	296	Canada	3.1 (2.7)	41.2 (12.5)
Bigras (2007)	Included 94% of all individuals with a history of sexual offense receiving a federal sentence in Québec between 1995–2000	452	Canada	3.0 (2.5)	42.7 (12.0)
Hanson et al. (2007, 2015)	Prospective study followed individuals with a history of sexual offense on community supervision between 2001–2005 in all Canadian provinces and territories	710	Canada	2.6 (2.4)	41.6 (13.3)
Lehmann et al. (2013)	Included 87% of all individuals with a history of sexual offense reported to the Berlin state police during the years 1994–2001 for a violent or abusive sexual offense	932	Germany	2.6 (1.7)	32.5 (10.4)
Study 2					
Blais and Bonta (2015)	Higher risk sample that includes individuals flagged under the NFS between 2004–2008 and individuals either designated as dangerous offenders or long-term offenders between 2006 and 2008	371	Canada	4.7 (2.1)	40.7 (11.4)
Hanson et al. (2007, 2015)	Prospective study followed individuals with a history of sexual offense on community supervision between 2001–2005 in all Canadian provinces and territories	710	Canada	2.6 (2.4)	41.6 (13.3)

*Note. M* refers to means, and *SD* refers to standard deviations.

#### Measures

The relevant measure for Study 1 was BARR-2002R (Babchishin et al., 2016; scoring sheet available in the online Supplemental Materials, Supplementary Figure S1), an actuarial risk scale for assessing general and violent (including sexual) recidivism among men with a history of sexual offending. BARR-2002R comprises age at release and the general criminality subscale of Static-2002R (Helmus et al., 2012) that includes items assessing prior involvement with the criminal justice system, community supervision violation, and history of nonsexual violence. BARR-2002R scores range from −2 to 8 and predict nonsexual violent (AUC = .74), any violent (AUC = .73), and general recidivism (AUC = .76; all estimates based on the random effects model) significantly better than the Static-2002R and Static-99R total scores in the development sample (Babchishin et al., 2016; see also Jung et al., 2018; Jung & Wielinga, 2019). BARR-2002R was also associated with other risk assessment tools designed to predict general recidivism and predicted general and violent recidivism just as well as measures specifically designed for these outcomes (Babchishin et al., 2016).

#### Analytical Strategy

In order to align BARR-2002R scores with the Justice Center’s Five-Level system, we computed a number of numerical indices. First, we computed the 2-year expected general recidivism rates using the procedures outlined by Hanson et al. (2017a). This involved a meta-analysis of logistic regression estimates using the routine, Canadian samples (*N* = 1,458, *k* = 3). The German sample was excluded for this analysis given that there were significant differences in the distribution of scores compared to the Canadian samples. In these analyses, BARR-2002R was centered on the median value of 2 (representing those scoring in the middle of the risk distribution). Additional information on the meta-analysis of the logistic regression coefficients for estimating the 2-year recidivism rates can be accessed online through the Supplemental Materials (Supplementary Tables S1 and S2).

We also computed hazard ratios for the expected 2-year general recidivism rates using a Cox regression survival analysis. As indicated in the procedures outlined by Babchishin et al. (2012), the samples were entered as strata to control differences in recidivism rates and the differences in the shape of survival function across the samples; we also included all routine samples (including the German sample; *N* = 2,390 *k* = 4). In order to identify the midpoint category needed to specify the Average Risk level, we used the median 2-year any recidivism rates of individuals with general offending as opposed to using a sample of individuals with a history of sexual offenses. Individuals with a history of sexual offending tend to score lower on general criminality than those individuals with no history of sexual offending; consequently, the midpoint is better represented by reference to the general population of individuals in the criminal justice system, not individuals with a history of sexual crime. The current sample represented men under some form of community supervision in Ontario, Canada (*N* = 16,782; Wormith et al., 2015). The 2-year general recidivism rate for this sample was 21.6%. In order to further inform the boundaries of the Five-Level system, we also computed the standard error of measurement (SEM) as $S\sqrt{1-r_{xx}}\;$ (Lord & Novick, 1968, p. 59), where *S* is the *SD* of BARR-2002R (based on the routine development samples, *k* = 4, *N* = 2390), and *r*_*xx*_ is the interrater reliability of the Static-2002R, used to infer BARR-2002R reliability given the overlap in items (Haag, 2005). The SEM for BARR-2002R was 0.88 (SEM = 2.20 $\sqrt{1-.84}$ ).

### Results

Table 2 provides the risk levels, following the Justice Center’s Five-Level system, for BARR-2002R, as well as the percentile ranks and 2-year general (any) recidivism rates. The following steps were used to align the total scores with the Five-Level system. In order to identify the middle level, we first used the logistic regression parameters (from meta-analysis) to compute 2-year general (any) recidivism rates for each BARR-2002R score. Using these recidivism estimates, we identified the BARR-2002R score associated with a 2-year expected general (any) recidivism rate that matched the 2-year any recidivism rate of 21.6% from the representative sample of individuals with general offending from Wormith et al. (2015). This procedure identified a BARR score of 4 as the closest value with an estimated recidivism rate of 21.2% compared to 21.6%. To account for measurement error for BARR-2002R (SEM of BARR-2002R = 0.88), this middle level or Level III was expanded up one unit and down one unit (scores from 3 to 5). This level included 40.7% of the sample of individuals with a history of sexual offending. Next, we found the level equivalent to the Justice Center’s Level I which has an expected general recidivism rate of less than 5% over 2 years. Based on the recidivism rate table, this level was associated with the three lowest values of BARR-2002R (−2 and 0).

**Table 2.** Standardized Risk Levels for the BARR-2002R in Study 1.

	Level		Percentiles		Risk ratio^a^	Predicted 2-year Recidivism rate^b^	Lower CI	Upper CI
BARR-2002R Score	Number	Name	Same Score	Cumulative Midpoint Average				
−2	I	Very low risk	7.0	3.5	0.09	1.3	0.8	2.2
−1	I	Very low risk	1.4	7.6	0.13	2.2	1.4	3.3
0	I	Very low risk	14.0	15.3	0.20	3.5	2.5	5.0
1	II	Below average risk	14.0	29.3	0.30	5.7	4.3	7.5
2	II	Below average risk	20.8	46.7	0.44	9.0	7.3	11.1
3	III	Average risk	9.4	61.8	0.67	14.0	12.1	16.3
4	III	Average risk	10.4	71.8	1.00	21.2	18.9	23.6
5	III	Average risk	9.6	81.8	1.50	30.6	27.8	33.6
6	IV	Above average risk	7.8	90.5	2.25	42.0	37.8	46.4
7+	IV	Above average risk	5.6	97.1	3.37	54.4	48.6	60.1

*Note.* Same score = percent with the same score. Cumulative midpoint average is the percentile value halfway between the percentiles calculated from (a) the proportion of the sample below the score and (b) the proportion with same score or lower (Crawford et al., 2009). CI = 95% confidence intervals. The midpoint level (III) was selected based on the expected 2-year recidivism rate that matched that of the median recidivism rate of an unselected sample of 16,782 male individuals in corrections in Ontario (2-year recidivism rate for the median LSI-OR score of 11 = 21.6%; Wormith et al., 2015).

^a^Risk ratios based on Cox regression coefficients derived from entering the raw BARR-2002R scores (*ß* = .4051; *SE* = .0225), with sample as strata (*k* = 4, *n* = 2,390, *n*_recidivists_ = 609, 1 case censored before event).

^b^Recidivism estimates based on routine Canadian samples (*N* = 1,458, *n*_recidivists_ = 247, *k* = 3) and a weighted fixed-effect *B*_*1*_ of .4971 (*SE* = .0388), a weighted fixed-effect *B*_*0*_ of −2.3094 (*SE* = .1182; centered on the median BARR-2002R value = 2), and a median correlation of the estimates of −.818. Recidivism estimates are not presented for a score of 8 (*n* = 6). See Online Supplemental Material for meta-analytical results (Supplementary Table S1).

We then captured the boundaries of the II and IV risk levels using +/− one treatment effect, the same procedure used by Hanson et al. (2017a). More specifically, the change from Level III to the next lowest and highest levels (levels II and IV) should be associated with the average treatment effect that has been reported in meta-analyses of evidence-based correctional programming. Based on different meta-analytic reviews (e.g., Andrews et al., 1990; Hanson et al., 2009), this treatment effect is estimated to be equivalent to an odds ratio of 0.70. This indicates that the lower boundary for Level III should be equivalent to an odds ratio of 0.70, while the upper boundary for Level III should be equivalent to an odds ratio of 1.43.

Combining the newly created Level III specifications with the boundaries already determined for Level I (scores of −2, −1, and 0), placed Level II as encompassing scores of 1 and 2; this level also had less than half the expected recidivism rates of Level III. Level IV was subsequently associated with a score of 5 or higher and had more than twice the expected recidivism rates of Level III. We did not find a Level V in BARR-2002R; as per the Justice Center’s recommendation, this level would have a 2-year recidivism rate of 85% or higher. The highest score on BARR-2002R was associated with a predicted 2-year general recidivism rate of 54% (95% CI = [49–60%]). The labels for the risk levels were modeled after the Static-2002R levels for sexual recidivism risk (Hanson et al., 2017a). Most individuals in the four development samples fell into Level II (31.9%; *n* = 762) and Level III (40.7%; *n* = 973), with few in the extreme risk levels (14.9%, *n* = 356 in Level I and 12.5%, *n* = 299 in Level IV). Five- and 10-year recidivism estimates for each individual BARR-2002R score are available online in the user manual (Babchishin et al., 2013).

### Discussion

It was possible to assign individuals to four of the five risk levels in the Justice Center’s Five-Level Risk and Needs System based on BARR-2002R scores. The risk levels substantially shared the same meanings in terms of recidivism rates and relative risk as the Justice Center’s originals. Unlike the Justice Center’s Five-Levels, but similar to Kroner et al. (2020) and Hanson et al. (2017b), BARR-2002R was not able to identify a group having expected general recidivism rates of 85% after 2 years. This could be a limitation of BARR-2002R. Given that BARR-2002R items are relatively simple variables based exclusively on criminal history records, someone can score in the high range by presenting with only a few risk factors (e.g., being young, having priors that include general violence); it is not surprising that it was unable to discriminate within the Above Average risk level. BARR-2002R may need items that more effectively distinguish between individuals at the highest levels of recidivism risk (think *item difficulty* from Item Response Theory; Embretson & Reise, 2013; Thomas, 2011). Examples of questions that could potentially separate out Level V from Level IV are the following: (a) number of institutional infractions during the past 3 months, (b) total time served in maximum security settings, and (c) more than one conviction for violence against institutional staff. These items may be useful in differentiating Level V from Level IV because they are plausible indicators of the very high end of the general criminality construct; however, they are presented as suggestions only and would need to be empirically tested prior to use in applied assessments. It is also possible that the inclusion of putatively dynamic factors, such as general criminality items in the STABLE-2007 and protective factors (Thornton, 2013), could provide a better estimate of the risk level and better identify those at the extreme levels. Future research should examine whether the combination of risk tools provides more accurate estimates of the risk levels hypothesized by the Five-Level system.

It could also be a limitation of the standardized risk levels, in that they could be proposing a conceptual level (Level V) that may not be found in nature or, at very least, not found with sufficient frequency to justify inclusion in a standardized set of risk levels. The distinction between Level IV and Level V is at the very high end of risk, and would most likely be useful in samples preselected to already be above average risk (e.g., high security settings), and over longer follow-up times. Nevertheless, Coulter et al. (2019) did identify 4.1% of a New Zealand community sample (*N* = 440) as Level V for the Roc*RoI (Bakker et al., 1999) based on expected recidivism rates of over 85% within 2 years. In contrast, Kroner et al. (2020) did not identify any individuals as Level V in two large, US community samples (*N* = 24,936 and *N* = 36,303). The lack of a Level V may also be attributed to the current sample being exclusively individuals with a sexual offending history, who tend to have lower general (overall) recidivism rates than individuals convicted of other crimes (Stewart et al., 2019).

Further research is needed to determine the frequency with which risk tools can identify Level V in diverse samples. If Level V is seldom found in large development samples, the threshold for Level V may be too high. Ideally, the threshold between Level IV and Level V would identify meaningful psychological differences between the groups. In the current model, Level IV individuals are expected to benefit from intensive treatment, whereas Level V individuals may not currently have the psychological readiness to benefit from rehabilitation efforts. The Five-Level system asserts that such psychological features are associated with recidivism rates of 85% or higher; however, this is an assertion awaiting empirical evidence. Evaluators using BARR-2002R should avoid equating high scores on BARR-2002R with extremely high risk for general recidivism.

We also found most individuals in our development samples fell into Level II (32%) and Level III (41%), with few in the extreme risk levels (15% in Level I and 12.5% in Level IV). Other studies applying the Five-Levels to general offending samples (rather than sexual offending samples) found a higher risk distribution, with most of their nonsexual offending sample scoring in Level III and IV on general risk tools. The New Zealand community corrections study found that most individuals fell into Level III (21%) and IV (54.5%) when using their tool, the Roc*RoI (Coulter et al., 2019). The two US community samples also found most individuals fell into Level III (40% in Sample 1, 19% in Sample 2) and Level IV (32% in Sample 1, 71% in Sample 2; Kroner et al., 2020). This is not surprising, as men with sexual offenses typically score lower on general criminality than men with nonsexual offenses (e.g., Craig et al., 2006).

We anchored the middle distribution to the general (any) reoffending rates of the general offending population so that BARR-2002R risk levels would have similar meaning to other risk tools for general criminality. This was a decision. We could have used the rates of general (any) reoffending for men with sexual offenses, but this would have resulted in risk levels that would not be expected to translate to other general offending tools following the Five-Level system. In other words, it would not allow us to compare BARR-2002R Five-Levels to the Five-Levels generated for other risk tools designed to assess risk for general (any) recidivism, as the anchor point would be substantially different (defined as the median recidivism rate for men with general offending vs. men with sexual offending).

One benefit of the standardized risk levels is that they can bring attention to findings that would not be otherwise obvious. For example, this study identified a substantial group of individuals with a history of sexual offending who present a very low risk for general recidivism (14.9% of the sample had an expected 2-year general recidivism rate of less than 5%). Such individuals are unlikely to benefit from correctional programming designed to reduce their risk of general criminal recidivism. Some of these individuals, nonetheless, may benefit from sex crime specific interventions. Using the standardized risk levels, determining the need for which type of intervention could be guided by considering separate assessments of the risk for general recidivism and for sexual recidivism. Although sexual recidivism is included in the BARR-2002R definition of any recidivism, the seriousness of sexual crime may justify intervention even when the observed sexual recidivism rates are relatively low (e.g., less than 10% after 5 years; see Hanson et al., 2017a).

## Study 2

### Purpose

Study 1 found that four of the five risk levels can be applied to BARR-2002R. It is, however, important to demonstrate that each of the defined levels also has the intended meaning. For example, is the risk profile of individuals placed within Level I similar to those proposed by the Five-Level system? What risk-relevant constructs differentiate individuals in Level I compared to those in Level II? In establishing the construct validity of the created levels, two convenience samples were utilized that contained a large number of variables that could be used for this purpose. The National Flagging System (NFS) sample represented a higher risk sample from the Canadian federal prison service, whereas the DSP represented a community sample who would be considered average risk for those with a sexual offending history in Canada. Although the average risk community sample was included in the samples used to develop the risk levels in Study 1, there was no overlap in the variables used in establishing the construct validity of the levels in Study 2. The items and risk scales available for each sample also differed. A direct comparison of the construct validity across the samples was therefore not possible. The important aspect of these analyses was to include both a higher risk and routine sample in order to assess potential differences in construct validity.

#### Hypotheses

It was expected that the risk profiles of individuals within each level would be consistent with the profiles provided in the original Five-Level descriptions (Hanson et al., 2017b). This congruency was expected to support an initial test of the construct validity of the Five-Levels. It was also expected that individuals within each risk level would not only share statistical indicators of risk, but also similarities in well-established constructs related to risk for individuals with a history of sexual offending. More specifically, it was expected that indicators of antisocial tendencies would increase from Level I to Level IV. By contrast, it was expected that indicators of sexual criminality would only weakly differentiate individuals placed in the different risk levels due to the fact that these indicators are not evenly distributed across individuals with sexual offenses against children versus adults (Whitaker et al., 2008) and that they are not consistently predictive of general offending (Hanson & Morton-Bourgon, 2009). Finally, individuals across risk levels should not differ on non-criminogenic domains under the Five-Level system (e.g., major mental illness).

### Method

#### Samples

*National Flagging System (NFS;* *Blais & Bonta, 2015**).* The first sample comprised 371 adult males who had been convicted of a sex offense in Canada and identified as high risk. In total, 244 individuals were flagged under the NFS between 2004 and 2008, and the remaining 127 individuals were either designated as dangerous offenders (*n* = 40) or long-term offenders (*n* = 87) between 2006 and 2008. This is the same sample that was utilized to validate the creation of BARR-2002R in a separate study (see Babchishin et al., 2016). The average age at release of the sample was 40.7 (*SD* = 11.4) and the average BARR-2002R score was 4.7 (*SD* = 2.1). Given the nature of the sample selection, the overall sample scored high on several well-established risk-relevant measurers including the PCL-R (*M* = 21.8, *SD* = 8.2) and the Static-2002R (*M* = 6.3, *SD* = 2.5).

*Dynamic Supervision Project (DSP;* *Hanson et al., 2007**;* *2015**).* Given the high-risk nature of the NFS sample, a separate, average risk sample of 710 individuals supervised in the community for a sexual offense was also utilized to evaluate the construct validity of the risk levels. This prospective study followed individuals on community supervision between 2001–2005 in all Canadian provinces and territories, and two US states. For the current study, only the Canadian samples were considered (*N* = 710) due to quality issues with the recidivism information of the US samples, with the highest numbers coming from New Brunswick (23.4%), Ontario (17.5%), British Columbia (16.5%), and Newfoundland (11.5%). The average age at release was 41.6 (*SD* = 13.3). The average BARR-2002R score was 2.6 (*SD* = 2.4).

#### Measures From the Higher Risk Sample Dataset (National Flagging System, *n* = 371)

*Level of Service/Case Management Inventory (LS/CMI;* *Andrews et al., 2004**).* The LS/CMI is an actuarial risk assessment tool that assesses the risk of general recidivism among adults. In addition to using total LS/CMI scores in the analyses, the domain score for criminal history was used as an indicator of antisocial tendencies for the higher risk sample along with the following individual items from the Introduction section and the Study 1 section: age at first conviction, never employed, diagnosis of psychopathy or antisocial personality disorder, institutional punishment, supervision failure, and failure in treatment. These items were chosen to represent the constructs included in the Five-Levels (i.e., risk for recidivism, treatment and supervision recommendations, and prognosis should treatment be provided). In order to examine indicators of mental illness and general functionality, the following items were also taken from the Introduction section and the Study 1 section for the higher risk sample: major mental illness, suicide attempt or ideation, dissatisfaction with marital relationship (or equivalent), financial difficulties, learning disability, and physical disability. For all items, higher scores indicated more problems.

*Psychopathy Checklist-Revised (PCL-R;* *Hare, 2003**).* The PCL-R is a construct rating scale designed to assess the personality and behavioral features of psychopathy among adults. The scale includes 20 items scored on a 3-point scale (0, 1, and 2) with total scores ranging from 0 to 40, with higher scores representing higher psychopathic tendencies. PCL-R scores were taken directly from the NFS files of the higher risk sample.

*Static-99R* (Hanson & Thornton, 2000). Static-99R is a 10-item actuarial measure that assesses recidivism risk of adult males with a history of sexual offending. The items are identical to Static-99 (Hanson & Thornton, 2000) with the exception of updated age weights (see Helmus et al., 2012). Scores range from −3 to 12, with higher scores representing a higher risk to sexually reoffend. Static-99R was assessed in the higher risk sample.

#### Measures From the Community Sample (Dynamic Supervision Project, *n* = 710)

*STABLE-2007* (Hanson et al., 2007)*.* The STABLE-2007 is a measure of risk-relevant factors relevant for the treatment and supervision of adult males charged or convicted of a sexually motivated offense. Total scores can range from 0 to 26, with higher scores indicating more criminogenic issues. In this study, we calculated the percent of individuals who scored a 1 or a 2 (representative of at least some elevation on the risk factors; see Hanson et al., 2017a) on the following items of the STABLE-2007: sexual preoccupation, lack of concern for others, number of negative social influences, cooperation with supervision, impulsive acts, hostility, and poor cognitive problem solving. As well, we examined three items from STABLE-2000, and earlier version of STABLE-2007 (Hanson et al., 2007): entitlement for sex, attitudes tolerant of sexual offending against adults, and attitudes tolerant of adult-child sex. These items were chosen because they best represented the overall constructs of Sexual Criminality and Antisocial Tendencies. The scores were only available for the average risk community sample.

*Screening Scale for Pedophilic Interests (SSPI;* *Seto & Lalumière, 2001**).* The SSPI consists of four items (male victim, unrelated victim, 2+ victims, and victim aged 11 or younger) and is intended to measure sexual interest in children among males who have committed a sexual offense against at least one child victim. Total scores range from 0 to 5, with higher scores indicating more pedophilic tendencies. The SSPI was computed from existing variables within the average risk community sample dataset as part of a separate study of the SSPI’s construct and predictive validity (see Helmus et al., 2015). We calculated the percent of individuals scoring 3 or higher based on findings that these scores represent a clinically relevant cut-score for the diagnosis of pedophilia (Brankley, 2019).

#### Procedure

In order to examine the construct validity of the four levels defined for BARR-2002R, individual risk-relevant variables were categorized into two of the three broader domains identified for sexual recidivism risk, namely, general criminality/antisocial tendencies, which was available for both the higher risk and average risk samples, and sexual criminality which was available for the average risk sample (Brouillette-Alarie et al., 2016, 2018; we were unable to assess the third domain of youthful stranger aggression). The broader domain of antisocial tendencies included individual risk-relevant items such as age at first conviction, never employed, and supervision failure in the higher risk sample; in the average risk community sample, antisocial tendency variables included lack of concern for others, impulsive acts, and hostility/grievance. In the higher risk sample, we were also able to consider how individuals from each level scored on relevant risk assessment scales and items that tapped into a non–risk-relevant domain such as the presence of mental illness and suicide attempts or ideation which we labeled General Functionality. We categorized mental health variables as non–risk-relevant based on large meta-analyses demonstrating either negative relationships or non-significant relationships between these variables and general and violent recidivism (Bonta et al., 2014). In the average risk sample, sexual criminality items included sexual preoccupation and attitudes tolerant of offending against children and adults.

All variables taken from the NFS higher risk sample were originally coded by a team of four coders; reliability estimates were calculated for 40 files and all original variables were good to excellent (ICC_A,1_ range: .65–1.00; kappa range: .66–1.00; Blais & Bonta, 2015). The LS/CMI and Static-2002R were coded in full using the file information, while the PCL-R score was taken directly from the files. Variables from the DSP average risk sample were coded by a sample of probation and parole officers as part of a large project examining the community supervision of individuals with a history of sexual offenses (Hanson et al., 2007). All officers underwent training on the scoring of each scale. To calculate rater reliability, 92 cases were rescored by 2 out of 7 expert raters; reliability estimates were good to excellent for the variables examined in the current study (ICC range: .66 to .95; total STABLE score ICC = .89).

#### Analytical Strategy

For continuous variables, means and *SD*s were calculated; for categorical variables, percent of individuals that were coded as having the risk factor were calculated. Sample sizes varied for each BARR risk level; however, in order to be included, the variable must have had at least ten cases per level. Although we were mostly interested in the congruence between the profiles of the individuals within each level and those provided by the original Five-Levels (descriptions in Appendix 1), we also calculated an index of the strength in association between the Five-Levels and each variable of interest. We ran a series of polychoric and polyserial correlations in Stata SE (Version 16). Polychoric and polyserial correlations estimate the relationship between two theorized normally distributed, continuous latent variables from observed variables that are either both ordinal variables (polychoric) or one ordinal and one interval/continuous (polyserial; Flora & Curran, 2004; Holgado-Tello et al., 2010). Polychoric and polyserial correlations were selected as opposed to Pearson correlations because applying Pearson correlations to ordinal data leads to restricted correlation coefficients and thereby provides less accurate estimates of the association compared to polychoric and polyserial correlations (Brown, 2006; Holgado-Tello et al., 2010). In interpreting the correlations, positive values indicate that the risk factor increases from the lowest to highest risk level, whereas negative values indicate that the risk factor decreases across the levels. Smaller values indicate that there is no clear progression across the Five-Levels.

### Results

#### Construct Validity Among a Higher Risk Sample

Table 3 presents the risk profiles for individuals within each identified risk level for the higher risk sample. Other than risk scale total scores, all of the variables refer to individual items on the LS/CMI. Level IV (Above Average Risk) had elevated scores on the LS/CMI (*M* = 30.9; *SD* = 6.8), Static-99R (*M* = 6.4; *SD* = 1.8), and PCL-R (*M* = 26.0; *SD* = 5.3). Level IV individuals also had a pervasive and sustained involvement in crime based on their LS/CMI criminal history score (*M* = 6.9; *SD* = 0.9; highest possible score on this scale is 8) and age at first conviction (*M* = 17.0; *SD* = 3.1). They also had serious treatment and management issues. In terms of general functioning, 82.2% presented with dissatisfaction with marital relationship and 84.9% had financial difficulties. Fewer had non–risk-relevant constructs such as mental illness and presence of disabilities. Overall, these individuals matched the expected risk profile based on the information within the Five-Level description (see Appendix 1).

**Table 3.** Construct Validity of the Standardized Risk Levels in a Canadian Sample of Individuals Flagged as Higher Risk for Study 2.

	I	II	III	IV	*r*
	*M* (*SD*, *n*)/% (*n*/*N*)	*M* (*SD*, *n*)/% (*n*/*N*)	*M* (*SD*, *n*)/% (*n*/*N*)	*M* (*SD*, *n*)/% (*n*/*N*)	
Risk Assessment Scales					
LS/CMI	14.9 (8.6, 15)	17.3 (7.6, 34)	25.4 (7.8, 104)	30.9 (6.8, 117)	.61
Static-99R	2.4 (1.5, 10)	4.2 (2.5, 19)	5.5 (2.0, 71)	6.4 (1.8, 82)	.49
Antisocial Tendencies					
PCL-R	—	13.8 (5.0, 14)	21.0 (8.4, 59)	26.0 (5.3, 57)	.51
Criminal history	2.5 (1.2, 20)	3.8 (1.7, 40)	6.0 (1.5, 124)	6.9 (0.9, 144)	.71
Age at first conviction^a^	44.1 (14.7, 20)	31.4 (12.2, 44)	22.0 (6.8, 144)	17.0 (3.1, 161)	.68
Never employed	5.3 (1/19)	7.9 (3/38)	20.9 (27/129)	55.4 (77/139)	.59
Psychopathy/APD	11.8 (2/17)	12.5 (5/40)	47.0 (62/132)	67.3 (101/150)	.52
Institutional punishment	20.0 (4/20)	26.8 (11/41)	64.6 (82/127)	87.3 (131/150)	.63
Supervision failure	10.0 (2/20)	41.9 (18/43)	88.8 (127/143)	98.1 (158/161)	.81
Failure in treatment	33.3 (6/18)	31.6 (12/38)	65.4 (87/133)	81.3 (122/150)	.47
General Functionality					
Major mental illness	16.7 (3/18)	19.5 (8/41)	19.3 (26/135)	23.8 (36/151)	.09
Suicide attempt/ideation	42.1 (8/19)	48.8 (20/41)	51.9 (70/135)	54.4 (81/149)	.08
Dissatisfaction with marital	60.0 (6/10)	55.6 (15/27)	62.5 (60/96)	82.2 (88/107)	.32
Financial difficulties	22.2 (4/18)	55.6 (20/36)	67.9 (72/106)	84.9 (107/126)	.48
Learning disability	10.5 (2/19)	5.0 (2/40)	21.6 (29/134)	26.1 (40/153)	.24
Physical disability	25.0 (5/20)	25.0 (10/40)	14.5 (20/138)	11.3 (17/151)	-.20

*Note.* LS/CMI = Level of Service/Case Management Inventory (Andrews et al., 2004); PCL-R = Psychopathy Checklist-Revised (Hare, 2003). Criminal history refers to the domain of the LS/CMI. The remaining variables under Antisocial Tendencies and General Functionality represent individual items of the LS/CMI. Correlation coefficient (*r*) represents polychoric and polyserial coefficients.

^a^Age-reversed scored in correlation analyses so that younger age is related to higher risk level.

Individuals within Level III (Average Risk) are meant to resemble the average justice-involved individual. Here again, we find good support for the levels created from BARR-2002R. Level III individuals had average scores on the LS/CMI (*M* = 25.4, *SD* = 7.8) and PCL-R (*M* = 21.0, *SD* = 8.4), although slightly elevated scores on the Static-99R (*M* = 5.5, *SD* = 2.0). Their overall risk profile was lower than Level IV individuals. The mean age at first conviction was 22 (*SD* = 6.8) and these individuals demonstrated stability in terms of employment. Although most had failed a supervision order in the past, just over one-third had never experienced institutional punishment or failed in treatment. There was also no appreciable increase in non–risk-relevant factors (e.g., mental illness, suicide, or disabilities) from Level III to IV. In fact, across all risk levels, there was only a small correlation between General Functionality variables and risk level placement (median *r* = .16). On the other hand, there was a moderate increase in Antisocial Tendencies variables across the risk levels (median *r* = .61).

Those in Level II (Below Average Risk) had lower than average scores on the LS/CMI (*M* = 17.3, *SD* = 7.6) and PCL-R (*M* = 13.8, *SD* = 5.0). Static-99R scores (*M* = 4.2, *SD* = 2.5) were still above average, suggesting sex crime specific problems. These individuals did not present with a long or entrenched criminal history with an average age at first conviction of 31 (*SD* = 12.2) and an average LS/CMI criminal history score of 3.8 (*SD* = 1.7). Approximately, one-quarter had a history of institutional punishment or failure in treatment; however, more than half had supervision failures, problems with marital relationships, and financial difficulties.

Unlike the other levels, the profile of individuals in Level I (Very Low Risk) did not fully match the description provided by the Five-Level system. In the original profile, Level I individuals are described as having few, if any, risk factors, and minimal prior contact with the criminal justice system. This description was supported by the relatively high mean age at first conviction (*M* = 44.1, *SD* = 14.7), high employment rates, and low rates of failure on supervision. This group of individuals, however, still presented with a mean LS/CMI score of 14.9 (*SD* = 8.6), indicating the potential for the presence of several risk-relevant factors. In fact, Kroner et al. (2020) found that a score of 14 on the LSI-R described Level III individuals in their samples. Furthermore, in the current sample, one in five had a history of institutional punishment, one-third had failed in treatment, and two-thirds had marital problems.

#### Construct Validity in the Dynamic Supervision Project Sample

Individuals were identified as having a clinically significant problem on items from the STABLE-2007 (defined as scores of 1 or 2) and on the SSPI (defined as a score of 3 or more; Brankley, 2019) in an average risk community sample (Table 4). A majority of individuals within Level IV presented with general antisocial tendencies (e.g., lack of concern for others and poor cognitive problem solving). These individuals also presented with problems related to sexual preoccupation, entitlement for sex, and attitudes supportive of sexual offending against adults. The overall rates of clinically significant problems for Level III were lower than for Level IV, but multiple problems were present, nonetheless. As expected, the differences between Level III and Level IV were not as evident for sexual criminality items, with 30.0–50.0% of the items being problematic for both risk levels. In general, Sexual Criminality items were weakly, and negatively, associated with increases in risk level placement (median *r* = −.17).

**Table 4.** Construct Validity of the Standardized Risk Level in a Canadian Community Sample for Study 2.

	I	II	III	IV	*r*
	% (*n/N*)	% (*n/N*)	% (*n/N*)	% (*n/N*)	
Sexual Criminality					
Sexual preoccupation	25.2 (29/115)	39.0 (80/205)	44.8 (77/172)	42.3 (33/78)	.17
Entitlement for sex	23.5 (27/115)	36.6 (75/205)	48.8 (84/172)	60.3 (47/78)	.32
SOA attitudes	17.4 (20/115)	28.8 (59/205)	40.5 (70/173)	50.0 (39/78)	.31
SOC attitudes	43.5 (50/115)	32.2 (66/205)	28.5 (49/172)	24.4 (19/78)	-.17
SSPI	28.6 (20/70)	32.8 (42/128)	30.6 (34/111)	47.5 (19/40)	.11
Antisocial Tendencies					
Lack of concern for others	32.2 (37/115)	33.7 (69/205)	37.8 (65/172)	69.2 (54/78)	.26
Negative social influences	22.9 (24/105)	34.5 (59/171)	38.4 (53/138)	49.2 (30/61)	.22
Lack of cooperation with supervision	15.6 (18/115)	20.5 (42/205)	37.6 (65/173)	53.8 (42/78)	.38
Impulsive acts	12.2 (14/115)	27.3 (56/205)	45.7 (79/173)	70.5 (55/78)	.50
Hostility/grievance	30.4 (35/115)	35.6 (73/205)	37.6 (65/173)	55.1 (43/78)	.18
Poor cognitive problem solving	39.1 (45/115)	48.3 (99/205)	64.7 (112/173)	89.7 (70/78)	.42

*Note.* SOA = sexual offending against adults. SOC = sexual offending against children. SSPI = Screening Scale for Pedophilic Interests (Seto & Lalumière, 2001). The remaining variables represent the percent of individuals scoring either a 1 or a 2 (vs. 0) on the following items of the STABLE-2007 (Hanson et al., 2007): sexual preoccupation, entitlement for sex, attitudes tolerant of sexual offending against adults and children, lack of concern for others, number of negative social influences, cooperation with supervision, impulsive acts, hostility, and poor cognitive problem solving. SSPI represents the percent of individuals scoring a 3 or above (vs. 0–2). Correlation coefficient (*r*) represents polychoric coefficients.

Based on the Five-Level descriptions, we would expect individuals in Level II (Below Average Risk) to require little intervention and to comply with orders of supervision, which was consistent with the finding that nearly 80.0% had had no issues with supervision compliance and nearly 75.0% have no issues with impulsivity. These individuals, however, still had risk-relevant indicators with a third or more having a lack of concern for others, hostility, and poor cognitive problem solving. In terms of sexual criminality, Level II individuals presented with fewer problems compared to Level III; nevertheless, approximately one-third had issues related to offending against children (32.2% having attitudes tolerant of offending against children and 32.8% having pedophilic interests).

Similar to what was found for the higher risk sample, individuals in Level I presented with more criminogenic needs than expected based on Five-Level descriptions. These included a lack of concern for others, hostility, and poor cognitive problem solving. The same was evident for the sexual criminality items. Despite relatively few individuals having attitudes tolerant of sexual offending against adults, approximately one quarter were scored as having issues with sexual preoccupation, entitlement for sex, and pedophilic interests. Just over 40.0% also presented with attitudes supportive of sexual offending against children.

### Discussion

Across both samples, the general antisociality risk profiles of individuals placed within BARR-2002R Level II (Below Average Risk), Level III (Average Risk), and Level IV (Above Average Risk) were substantially consistent with the profiles as originally defined by the Five-Level system (Hanson et al., 2017b). For example, on average, Level III individuals presented with several risk factors that should influence decisions concerning risk management (e.g., LS/CMI criminal history and institutional problems) and treatment compliance (e.g., STABLE-2007 impulsivity and poor cognitive problem solving). By contrast, individuals in Level II presented with fewer of these risk factors, while individuals in Level IV presented with more, as expected.

Unlike the general risk items, the presence of clinically significant scores on important dynamic factors for sexual recidivism and higher scores on the Static-99R was evident across all identified risk levels. The elevated Static-99R scores indicate that BARR-2002R risk levels were not particularly sensitive to the risk of sexual recidivism. For example, based on the standardized risk levels for the Static-99R (see Hanson et al., 2017a), scores of 1, 2, and 3 are considered average risk for sexual recidivism. In the high-risk sample, the average Static-99R score for BARR-2002R Level III individuals was over 5. The higher level of sexual criminality—as indexed by the Static-99R—could be due to the higher risk nature of the NFS sample; these individuals are considered to be preselected high risk (see Hanson et al., 2016) given that they have been flagged as potential candidates for preventative detention, primarily based on their unusually serious sexual offending history.

We mostly found that general functionality factors, such as major mental illness and suicide attempts/ideation, did not distinguish between the levels. There were a few exceptions; indicators of financial difficulties and dissatisfaction with marital status increased from Level I to Level IV. This is likely due to the fact that these items are tapping into established risk factors for general offending such as employment and interpersonal difficulties (Andrews & Bonta, 2010). Indeed, financial difficulties, and debt in particular, have been associated with criminal activity (van Beek et al., 2020).

The overall presence of sexual criminality items across the levels, however, was expected given that BARR-2002R was intended to measure general criminality, and that general criminality and sexual criminality are not highly correlated. The pattern of results can also be explained by assuming that the sample contained some portion of individuals who were high risk for sexual recidivism but low risk for general recidivism. This may seem like a contradiction; however, sexual offense recidivism is high severity and occurs over a long period of time. Consequently, a 5.0% recidivism rate after 2 years would indicate above average risk for sexual recidivism if all the offenses were sexually motivated (Static-99R score of 4, Above Average Risk, see Table 7 in Thornton et al., 2019). Regardless of the explanation, these findings reinforce the importance of considering separately the risk of sexual and the risk of general recidivism. Indeed, the lack of concordance between general and sexual criminality factors suggests that individuals would be expected to differ on the Standardized Risk Levels designed for general criminality than risk levels designed for sexual criminality.

We also did not find a group consistent with the lowest risk level comprised of prosocial individuals whose criminal involvement is an exception to otherwise well organized and productive lives. Although we identified individuals with very low recidivism rates (< 5% after 2 years), these individuals were not without criminogenic needs. This may indicate the need to revise the Five-Levels definitions by considering crime trajectories and, likely, by including changeable risk factors that are sensitive to change in criminal propensities. Very low recidivism rates can be found among prosocial individuals who are offending for the first time, as well as among individuals at the end of long criminal careers. The larger variability in age at first conviction in both Levels I and II provide some support for this possibility. Almost all individuals with a criminal history eventually desist from crime (Hanson, 2018). Even individuals who were once considered high risk will present no more than a minimal risk for recidivism should they remain crime free long enough (Bushway et al., 2011; Hanson et al., 2014, 2018). The reasons for desistance from crime are not fully known. Internal factors such as change in identity and external factors such as marriage and employment are thought to be important to the process of desistance (Laub & Sampson, 2009; LeBel et al., 2008). It is likely that the 40- or 50-year-old at the end of a long criminal career would have more difficulty in establishing the factors that promote desistance compared to the generally prosocial individual who made a mistake, despite both being objectively low risk for recidivism.

A potential limitation of the Five-Level system is that it implicitly assumes that effective rehabilitation requires reductions in criminogenic needs. For example, an individual may transition from Level III to Level II by distancing from negative peers, drinking less, and maintaining steady employment. An alternate model is that long-term vulnerabilities do not disappear; instead, individuals learn to manage them (Hogan, 2020; Olver et al., 2018). An individual may have an enduring propensity to become preoccupied with atypical, illegal sexual thoughts when stressed, but may also have learned effective ways to inhibit these impulses. Such individuals could be genuinely low risk for recidivism, but have a history and clinical presentation quite different from the prosocial individual who made an isolated mistake. Research on the treatment implications of the Five-Level system is needed. The Five-Level system assumes that successful correctional programming can result in individuals moving to lower levels, an assumption that awaits empirical testing.

### General Discussion and Recommendations

The current research indicated that the Five-Level system (Hanson et al., 2017b) summarizes and communicates much of the information provided by BARR-2002R scores. For all but BARR-2002R aficionados, the statement that an individual is below average risk for general recidivism would say more than stating that the individual’s BARR-2002R score was a 1. Furthermore, being placed in the Below Average risk category would communicate information concerning the expected recidivism rate, the number and severity of criminogenic needs, and, importantly, recommendations for effective correctional responses. The statistical indicators of risk required to align scores with standardized risk levels were relatively simple to calculate and provided matches to four of the five possible risk levels; we did not find BARR-2002R scores associated with the highest risk level reserved for individuals with expected two-year recidivism rates above 85%). We also found some preliminary evidence for the construct validity of most of the levels in two different Canadian samples, while identifying important limitations associated with populating the lowest risk level (Very Low Risk or Level I). Based on these findings, we can make several recommendations for research on standardized risk communication and the clinical implication of such research.

The goal of applying the standardized Five-Level system is to improve consistency in risk communication and to enhance the meaning of risk level labels. In doing so, the Five-Levels have the potential to enhance correctional responses to individuals based on their assigned level. The Five-Level system identifies relevant need and strength-based factors, provides a recommendation for intervention, and the expected prognosis should the appropriate correctional response be implemented. Although the Five-Level system is a promising advancement in risk communication, we were unable to identify Level V (with expected general 2-year recidivism rates over 85%) in Study 1. This is not entirely inconsistent with existing studies. Kroner et al. (2020) did not find Level V in their two large samples. Coulter et al. (2019), however, did identify a Level V group in a community sample in New Zealand. Further research is needed to determine the frequency with which risk tools can identify Level V in diverse samples and for different offending outcomes. Large, routine samples are required for this endeavor. Although we had four unique samples representing just over two thousand individuals, larger representative samples would always be helpful. If Level V is seldom found, the threshold for Level V may be too high, or the level may be unnecessary.

Study 2 was limited in the number and types of variables available to establish the construct validity of BARR-2002R levels. In fact, to date, we have only established that a small number of risk-relevant factors provide a risk profile consistent with the Five-Level descriptions. We have therefore provided preliminary evidence that the Five-Levels appear to correspond to the Risk and Need principles of the RNR model, although a fuller test of the Need principle would require assessment of change on the criminogenic needs. Furthermore, we have yet to assess the ability of strength-based factors to provide further construct validity to the different levels, nor has any research examined whether the recommended correctional responses would result in the hypothesized cascading down of levels. An important next step in validating the Five-level system is to assess changes in risk level placement over time, following appropriate correctional responses. Certainly, much work remains in establishing the construct and predictive validity of the Five-Level system.

Hanson et al. (2017a) provided preliminary evidence that the application of the Five-Level system could increase the concordance rate of two sexual recidivism risk scales (Static-99R and Static-2002R). Increased concordance has yet to be demonstrated for other tools (the study would require at least two risk tools that follow the Five-Level system). Given growing evidence for the discordance of risk categories, even among scales designed to assess the same outcome (e.g., Jung et al., 2013), further evidence of the Five-Level system’s ability to reduce discordance across risk measures would be beneficial for enhancing risk communication practices. This is especially true given that evaluators typically use a number of different tools within the same assessment (Blais & Forth, 2014; Viljoen et al., 2010), and that the inclusion of information from multiple risk scales can enhance predictive accuracy and calibration (Babchishin et al., 2012; Lehmann et al., 2013).

The results from these studies also highlighted the importance of considering the underlying constructs that inform risk assessments. The factors that are relevant for assessing general recidivism outcomes will not necessarily be the same as those for other recidivism outcomes. For example, despite being below average risk on indicators of general criminality, individuals can still present a meaningful number of risk-relevant factors of sexual criminality. Appropriate treatment planning therefore requires a consideration of a number of risk-relevant factors for each desired outcome. In addition, concordance rates between the Standardized Risk Levels for tools designed to assess general criminality and those assessing sexual criminality are not expected to be high given that these tools are assessing different risk-relevant constructs. In adopting the Five-Levels, researchers and evaluators should therefore carefully consider what the primary outcome of interest is and whether the tools selected are adequately assessing constructs relevant to that outcome (Hogan, 2020).

The existence of two dimensions of recidivism risk complicates the application of the Risk principle in Andrews et al. (1990) RNR model. Multiple dimensions of risk should not be a concern for therapists providing individualized treatment, who are accustomed to their clients having more than one area of concern. In many correctional settings, however, there are a limited suite of structured programs available, and case managers must decide which programs are appropriate for the case-at-hand. For example, case managers may decide to refer individuals to general offending programs if they are average (Level III) or above average risk (Level IV) for general recidivism and very low risk (Level I) for sexual recidivism. Conversely, some form of sex crime specific treatment could be recommended for individuals who are average (Level III) or above average risk (Level IV) for sexual recidivism. Although simultaneously considering sexual and general recidivism risk complicates program referral, it is not a new problem and has been already directly addressed by many correctional systems (e.g., see the National Correctional Program Referral Guidelines for the Correctional Service of Canada (2018)).

### Recommendations for Users of Brief Assessment of Recidivism Risk-2002R

BARR-2002R is a brief actuarial measure addressing a limited number of relevant risk factors that provides a more accurate estimate of the likelihood of general recidivism than Static-2002R and Static-99R for individuals with a history of sexual offending (Babchishin et al., 2016). That said, it is a screening tool. We expect that its primary utility will be with individuals who have already been scored on the full Static-2002R risk tool (i.e., those with a sexual offense conviction). Evaluators interested in a comprehensive examination of general criminality are encouraged to supplement BARR-2002R assessments with other validated risk assessment tools, such as the LS/CMI.

Given the results of both Study 1 and Study 2, we recommend that the Five-Level Risk and Needs System, along with risk ratios and absolute recidivism rates presented in the BARR-2002R user manual, be used when communicating the results of BARR-2002R. We caution that individuals falling within the lowest risk level (Level I) may still have risk-relevant factors that need to be addressed for their successful reintegration, despite having a low risk for general reoffending.

## Supplemental Material

sj-pdf-1-sax-10.1177_10790632211047185 – Supplemental Material for Improving Our Risk Communication: Standardized Risk Levels for Brief Assessment of Recidivism Risk-2002R Click here for additional data file. Supplemental Material, sj-pdf-1-sax-10.1177_10790632211047185 for Improving Our Risk Communication: Standardized Risk Levels for Brief Assessment of Recidivism Risk-2002R by Julie Blais, Kelly M. Babchishin and R. Karl Hanson in Sexual Abuse: A Journal of Research and Treatment

## Appendix

**Table A1.** Summary of the Five-Level Risk and Needs System (Adapted from Hanson et al. (2017b)).

Risk Level	Identifiable Needs/Strengths	Correctional Response	Prognosis
Level I	- Few needs; clear identifiable strengths	- Prison would be counter productive	- Offending risk is already so low, expect no change; expect to desist from crime completely
	- Low risk of any reoffending (less than 5%)	- Expected to comply with conditions/supervision	
Level II	- 1 or 2 needs (low severity); some identifiable strengths	- Long- term custody would be counter productive	- With proper response, will transition to Level I; desistance is likely
	- Low rate of any reoffending (average of 19% at 2 years)	- Expected to comply with conditions - Short- term interventions	
Level III	- Multiple needs (varying severity); have resources, but needs impede utilizing them	- Custody appropriate for short- term	- With proper intervention, expected to reduce reoffending
	- Moderate rates of any reoffending (average of 40%)	- Require more dosage of treatment (100–200 h)	- Risk of reoffending will be higher than general population
Level IV	- Many needs (chronic and severe); some resources but chronic barriers to access them	- Have a history of incarceration; require intensive community supervision; intensive and lengthy programming (200–300 h)	- With appropriate strategies, significant reductions in reoffending expected; even so, rate of reoffending likely to remain around Level III
	- Higher risk of any reoffending (average of 65%)		
Level V	- Most, if not all, of need areas present (chronic, severe, and longstanding); limited strengths/resources	- Custody is appropriate	- Reductions in reoffending slow and gradual (over decades)
	- High rates of any reoffending (average of 85%)	- Highly structured, intensive, lengthy treatment (over 300 h); occur in facilities prior to release	- Reoffending expected to remain high regardless
