# Supplementary material for: Improving Our Risk Communication: Standardized Risk Levels for Brief Assessment of Recidivism Risk-2002R
Source: Sex Abuse. 2021 Oct 20;34(6):667–98. doi: 10.1177/10790632211047185 (PMC9379389; doi:10.1177/10790632211047185)
Supplement: sj-pdf-1-sax-10.1177_10790632211047185 – Supplemental Material for Improving Our Risk Communication: Standardized Risk Levels for Brief Assessment of Recidivism Risk-2002R [file sj-pdf-1-sax-10.1177_10790632211047185.pdf]

Online Supplemental Materials

Table 1S

Meta-Analysis of the Logistic Regression Coefficients for Estimating 2-Year General Recidivism Rates for the BARR-2002R

	Fixed effect		Random effects				
	M (SE)	95% CI	M (SE)	95% CI	Q	I^2	Nrec/N (k)
B_1	0.4971 (.0388)	0.42, 0.57	0.4971 (.0388)	0.42, 0.57	1.70	0.00	247/1458 (3)
B_0 (raw)	-2.3094 (.1182)	-2.541, -2.0777	-2.6262 (.3704)	-3.3522, -1.9002	13.13***	84.8	247/1458 (3)
(%)	9.0	7.3, 11.1	6.7	3.4, 13.0			

Note. Nrec = number of recidivists. B_1 (the slope) is an estimate of relative predictive accuracy, or the average change in recidivism rates for each one-unit increase in risk scores, expressed as a log odds ratio. B_0 is an estimate of the base rate for general recidivism for individuals scoring in the middle of the risk distribution (centered on the median BARR-2002R value = 2). For ease of interpretation, the B_0 was transformed into probabilities (*p*), where $p = e^{\text{LOGIT}}/(1 + e^{\text{LOGIT}})$. The SEs, however, are in the original metrics (logits). Despite variability, no statistical outliers were found in B_0. *** p <.001

Table 2S

Two-Year Observed and Estimated General Recidivism Rates for BARR-2002R (based on fixed-effect meta-analysis results)

Score	Fixed Follow-up		Logistic Regression Estimates		
	Recidivists/ total	Observed Recidivism Rate (%)	Predicted Recidivism Rate (%)	95% CI	
-2	1/82	1.2	1.3	0.8	2.2
-1	0/17	0.0	2.2	1.4	3.3
0	6/179	3.4	3.5	2.5	5.0
1	10/179	5.6	5.7	4.3	7.5
2	27/251	10.8	9.0	7.3	11.1
3	15/156	9.6	14.0	12.0	16.3
4	33/174	19.0	21.2	18.9	23.6
5	39/153	25.5	30.6	27.8	33.6
6	54/150	36.0	42.0	37.8	46.4
7	59/111	53.2	54.4	48.6	60.1
8	3/6	50.0	-	-	-
Total	247/1,458	19.5			

Note. Recidivism estimates based on routine Canadian samples (N = 1,458, $n_{recidivists}$ = 247, k = 3) and a weighted fixed-effect B_1 of .4971 (SE = .0388), a weighted fixed-effect B_0 of -2.3094 (SE = .1182), and a median correlation of the estimates of -.818. Recidivism estimates are not presented for a score of 8 (n = 6).

Figure 1S

Coding form for the Brief Assessment for Recidivism Risk (BARR-2002R)

<u>**AGE**</u><br>**1. Age at Release**<br>18 to 34.9 = 2<br>35 to 39.9 = 1<br>40 to 59.9 = 0<br>60 or older = -2	
<u>**GENERAL CRIMINALITY**</u><br>**2. Any Prior Involvement with the Criminal Justice System**<br>No = 0<br>Yes = 1	
**3. Prior Sentencing Occasions For Anything:**<br>0-2 prior sentencing occasions for anything = 0<br>3-13 prior sentencing occasions = 1<br>14 or more prior sentencing occasions = 2	
**4. Any Community Supervision Violation:**<br>No = 0<br>Yes = 1	
**5. Years Free Prior to Index Sex Offence:**<br>• More than 36 months free prior to committing the sexual offence that resulted in the index conviction AND more than 48 months free prior to index conviction = 0<br>• Less than 36 months free prior to committing the sexual offence that resulted in the index conviction OR less than 48 months free prior to conviction for index sex offence = 1	
**6. Any Prior Nonsexual Violence Sentencing Occasion:**<br>No = 0<br>Yes = 1	

Note. Coding rules for items and norms for BARR-2002R are available on www.static99.org
